# Prevalence and risk factors of elevated alanine aminotransferase among Korean adolescents: 2001-2014

**DOI:** 10.1186/s12889-018-5548-9

**Published:** 2018-05-11

**Authors:** Ju Whi Kim, Kyung Jae Lee, Hye Ran Yang, Ju Young Chang, Jin Soo Moon, Young-Ho Khang, Jae Sung Ko

**Affiliations:** 1 Department of Pediatrics, Seoul National University College of Medicine, Seoul, South Korea; 2 Department of Health Policy and Management, Seoul National University College of Medicine, Seoul, South Korea

**Keywords:** Alanine aminotransferase, Prevalence, Fatty liver

## Abstract

**Background:**

An elevated alanine aminotransferase (ALT) level is a surrogate marker of non-alcoholic fatty liver disease (NAFLD), the most common liver disorder in adolescents. The majority of previous NAFLD studies in adolescents were performed in selected obese populations or had a cross-sectional design without a time-trend analysis. The purpose of this study was to estimate the prevalence and time trends of elevated ALT levels in a general adolescent population and to identify factors associated with ALT elevation.

**Methods:**

We analysed data of adolescent participants (aged 10–18 years) from the Korean National Health and Nutrition Examination Survey 2001–2014, a representative sample of the general population in South Korea. Suspected NAFLD was defined as ALT elevation (> 30 U/L) without hepatitis B surface antigen. In all statistical analyses, sampling weight- and design-based data were used.

**Results:**

ALT was elevated in 5.3% (standard error: 0.3%) of the study population of adolescent participants (*N* = 8455). No significant trends were found from 2001 to 2014 in the prevalence of elevated ALT among male and female adolescents. In multiple logistic regression analysis, elevated ALT was independently associated with sex (odds ratio [OR] male versus female 4.5; 95% CI, 3.3-6.2), obesity (OR 7.6; 95% CI, 5.3-11.0), and truncal obesity (OR 2.5; 95% CI, 1.8-3.5). Furthermore, male sex, obesity, truncal obesity and high household income level were associated with log-transformed ALT levels in multiple regression analysis.

**Conclusions:**

In Korean adolescents of both genders, the prevalence of elevated ALT levels was stable from 2001 to 2014. This study has revealed that sex, obesity, truncal obesity and household income level are associated with ALT elevation in adolescents.

## Background

Non-alcoholic fatty liver disease (NAFLD) is defined as the accumulation of fat in the liver in the absence of excessive alcohol consumption or other known liver pathologies [1]. Since the first cases of paediatric NAFLD were reported in 1983 [2], NAFLD has become one of the most common causes of chronic liver disease in children in the developed world, and its prevalence is increasing in the developing world [3, 4]. Paediatric NAFLD is associated with numerous serious health conditions, such as cardiovascular disease and metabolic syndrome, which includes dyslipidaemia and hypertension [3]. A recent systematic review based on 20 surveys of different populations reported that the prevalence of NAFLD in the general paediatric population is 7.6%, and there is no evidence that it has changed over time [1].

The majority of previous NAFLD studies in adolescents were performed in selected obese populations or had a cross-sectional design without a time-trend analysis. Expert Committee guidelines recommend the use of serum alanine aminotransaminase (ALT) levels to screen for NAFLD [5]. Despite controversies regarding the upper limit of normal (ULN) in children, an elevated ALT level is a common diagnostic criterion for NAFLD, especially in studies of the general population [3]. Liver biopsy, the gold standard for diagnosing NAFLD, is invasive, expensive and impractical as a population-level screening test. Ultrasound imaging, which is non-invasive and relatively inexpensive, is used for the diagnosis of NAFLD in the general population; however, this imaging method is not feasible for determining the NAFLD prevalence in a nationwide survey [1]. Based on ALT levels, the prevalence of NAFLD in adolescents in the United States increased over the past 20 years [5]; in Korea, the prevalence was 6.5% in the Korean National Health and Nutrition Examination Survey (KNHANES) 2007-2009 [6].

In addition, it has been proposed that factors other than obesity and metabolic syndrome are associated with NAFLD [4]. The prevalence of NAFLD among adults is higher in countries with a higher economic status; however, socioeconomic status is inversely associated with elevated ALT levels among adolescents in the United States [7]. There have been few studies of the associations between NAFLD and socioeconomic factors, such as household income level and parental education level. The purpose of this study was to estimate the prevalence and trend of elevated ALT among Korean adolescents and to identify risk factors, such as socioeconomic factors, to use for NAFLD screening using national data from 2001 to 2014.

## Methods

This study was based on the KNHANES administered by the National Centre for Health Statistics of the Korean Centres for Disease Control and Prevention (KCDC). The cross-sectional survey consisted of health interview, physical examination, and laboratory data from a complex multistage, stratified, clustered probability sample which is designed to be representative of the general Korean population [8]. The KNHANES was conducted beginning in 1998 to evaluate the health and nutritional statuses of adults and children in Korea. The first 3 KNHANES waves were conducted as triennial surveys: KNHANES I (1998), KNHANES II (2001), and KNHANES III (2005). To provide more current national statistics, it was converted to an annual survey from KNHANES IV (2007). Of the about 200,000 geographically defined primary sampling units (PSUs) for the entire country, 192 PSUs were drawn. A PSU consisted of 60 households on average, and 20 were selected as final target households through systematic sampling. Each KNHANES wave contains a new sample of approximately 10,000 individuals. At a mobile examination centre, the interview and physical examination are conducted by trained survey team, including health professionals and supporting staff. The response rate for the KNHANES is targeted at 75% in overall; as an example, the response rate for the 2011 KNHANES was 76.1% for the health interview and examination survey [8]. More information of the design and characteristics about the survey is available at the KNHANES website (https://knhanes.cdc.go.kr/knhanes/main.do).

We analysed the data for the adolescents (aged 10-18 years) from the KNHANES 2001-2014 (wave II-VI), a representative sample of the general Korean population. We excluded data from KNHANES wave I (1998), which had several limitations, such as overestimated rates of diabetes mellitus due to sampling error. To explain differential probabilities of selection and non-response, we included sample weights in the estimation procedures of the analyses. To analyse the annual prevalence and its trend, the weighted data were subsequently modified to represent the Korean population aged 10 to 18 years, as estimated by the Korean Census in 2005. KCDC obtained informed consent from all the participants, and the Institutional Review Board of the KCDC approved the protocol for the KNHANES.

### Definition of elevated ALT

In the KNHANES, serum samples were analysed at a central certified laboratory. Plasma concentrations of ALT and other parameters were measured by an auto-analyser. An elevated ALT level was defined as > 30 U/L, which is the value used in previous studies on adolescents [7]. We also conducted a repeat analysis using the 97.5th percentile for ALT (33 U/L for male adolescents and 25 U/L for female adolescents), as suggested by a recent report based on KNHANES data [6].

### Other variables

To determine factors related to ALT elevation, we used socio-economic information, demographic information and anthropometric measures as variables. The KNHANES health interview questionnaire consist of information provided by an adult respondent aged 19 years or older from a sampled household and contains household and individual components. We collected data on sex, residency (e.g., urban or rural), age (i.e., years), household income and parental education level to define the subgroup at high risk for ALT elevation. Quartiles of household income were provided by the KNHANES data. Parental educational levels were divided into middle school or lower, high school, and university or higher. We also merged the data for each parent to ascertain the parental education level, which has been included in the KNHANES protocol since 2007.

All health examinations were conducted by trained medical staff according to standardized protocols. Measurements of body weight, height, and waist circumference were conducted to the nearest 0.1 kg or 0.1 cm. Body mass index (BMI) was calculated as the ratio of weight in kilograms to height in metres squared (kg/m^2^). Using Korean National Growth Charts [9], we defined the cut-off point for obesity as a BMI at the 95th percentile or 25 kg/m^2^). The cut-off point for truncal obesity was defined as the 90th percentile.

Study subjects were excluded when they had hepatitis B surface antigen, did not have an available blood sample, or were missing data for covariates.

### Statistical analysis

Using the R statistical programming language, we generated figures for the time trend of elevated ALT prevalence and log-transformed ALT level [log_e_ALT] for each survey wave. *P* values for linear time trends were calculated using survey regression analysis (SAS, Proc surveyreg) after taking into account the primary sampling units, stratification, and sample weights for time trends. Odds ratios (ORs) and 95% confidence intervals (CIs) for the prevalence of elevated ALT were calculated by univariate survey logistic regression analyses using adjusted sample weights for the study population. Controlling for the factors that showed a significant association in univariate analyses, multiple logistic regression (SAS, Proc surveylogistic) was used to estimate the OR associated with an elevated ALT level. Subsequently, survey regression analysis was performed to estimate the associations among factors, with log_e_ALT as a continuous variable. Factors thought to contribute to log_e_ALT were analysed after adjusting for age and sex in a stepwise multiple linear regression. The sample weights were derived to allow the sample subjects to represent the general Korean population by explaining the complex survey design, survey non-response and post-stratification. Sample weights were based on the inverse of selection probabilities, and response rates adjusted for sex and age according to census controls. To reflect the differing probabilities of selection, non-response and non-coverage, these sample weights were included in the estimation processes for all analyses. All of the statistical analyses were conducted using SAS version 9.3 (SAS Institute, Inc., Cary, NC) with a significance level of *P* value < 0.05.

### Ethics statement

This study was exempted from Institutional Review Board (IRB) review because it contained open data and does not include any personally identifiable information (IRB No. E-1608-114-786).

## Results

**Fig. 1:**
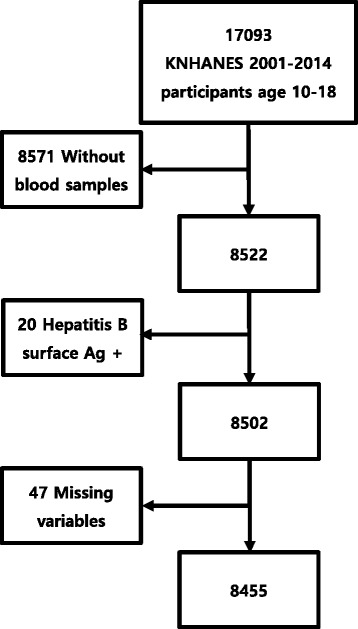
Inclusion and exclusion of the Korean National Health and Nutrition Examination Survey (KNHANES) reference population

**Fig. 2:**
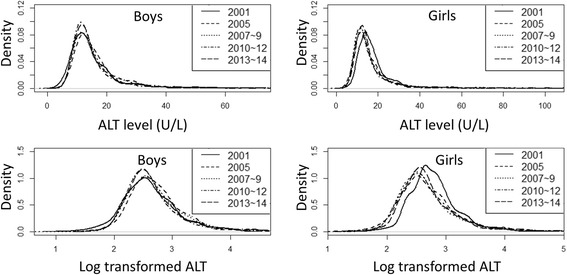
Density plots of the ALT level and log-transformed ALT in adolescents between 2001 to 2014 by gender

Of 17,093 adolescents who participated in the survey, 8522 provided blood samples and formed our study population. Adolescents who were positive for hepatitis B surface antigen (*n* = 20) were excluded. Additionally, we excluded 47 adolescents because of missing data for any of the factors, as required in the exclusion criteria. Figure 1 shows the flow chart of sample selection for this study. The weighted and standardized prevalence of elevated ALT levels (defined as ALT > 30 U/L) was 5.3%, and the median ALT level was 12.0 U/L (interquartile range, 10-16) in the study population. The prevalence of elevated ALT was 2.1% among female adolescents and 8.0% among male adolescents (*P* < 0.001). Density plots of the time trends of ALT levels are presented in Fig. 2. No significant trend was found in the prevalence of elevated ALT among male and female adolescents from 2001 to 2014 (Table 1). Suspected NAFLD was present in 2.6% of non-obese adolescents and 24.2% of obese adolescents. Using a higher ULN (defined as ALT > 40 U/L), the prevalence of elevated ALT levels was 2.8%. We repeated the analysis using gender-specific ULNs (> 33 U/L for boys and 25 U/L for girls), and the resulting prevalence was 5.4%.

**Table 1:** Time trend for elevated ALT prevalence by gender: 2001-2014

Prevalence % (SE^a^)	Wave II (2001)	Wave III (2005)	Wave IV (2007-9)	Wave V (2010-2)	Wave VI (2013-4)	Total	P for trend**
ALT> = 30 M	6.9(1.1)	6.4(1.2)	9.5(1.0)	7.8(0.9)	7.9(1.1)	8.0(0.5)	0.1563
ALT> = 30 F	1.6(0.6)	2.0(0.7)	1.9(0.4)	1.9(0.5)	1.4(0.5)	2.1(0.2)	0.9343
Total	4.4(0.7)	4.4(0.8)	6.0(0.6)	5.1(0.5)	4.8(0.7)	5.3(0.3)	0.2694

^a^*SE* standard error

**P-value from survey regression analysis

In the univariate logistic regression analysis, elevated ALT levels were significantly associated with male sex, age, residency, obesity and truncal obesity. In the age- and sex-adjusted analysis, residency, obesity and truncal obesity were significantly associated with elevated ALT levels. In the multiple logistic regression analysis, male sex, obesity, and truncal obesity were independent predictors of elevated ALT (Table 2). A comparison of the adolescents whose parents were college graduates and the other groups showed that neither maternal nor paternal education level was significantly associated with elevated ALT.

**Table 2:** Logistic regression analysis by elevated alanine aminotransferase

	Univariate		Age, sex adjusted		Multivariate	
variable	OR (95% C.I.)	*P*-value	OR (95% C.I.)	*P*-value	OR (95% C.I.)	*P*-value
Sex	4.5(3.4-6.0)	< 0.001	4.5(3.4-6.0)	< 0.001	4.5(3.3-6.2)	< 0.001
Age (year)	1.1(1.0-1.2)	< 0.001	1.1(1.0-1.2)	< 0.001	1.1(1.0-1.1)	0.0684
Town (urban vs. rural)	1.4(1.0-2.0)	0.036	1.4(1.0-2.0)	0.041	1.0(0.9-1.9.)	0.1422
Obesity	15.2(11.8-19.7)	< 0.001	11.2(8.8-14.4)	< 0.001	7.6(5.3-11.0)	< 0.001
Truncal obesity	9.1(7.2-11.5)	< 0.001	11.0(8.6-14.1)	< 0.001	2.5(1.8-3.5)	< 0.001
Income	1.1(0.8-1.6)	0.5214	1.1(0.6-1.7)	0.675	–	–
Edu^a^ F (quartile)	0.8(0.7-1.0)	0.63	0.9(0.7-1.1)	0.1786	–	–
M (quartile)	0.9(0.7-1.0)	0.1312	0.9(0.7-1.1)	0.3564	–	–

*Parental education level was included in the KNHANES protocol starting in 2007

**Fig. 3:**
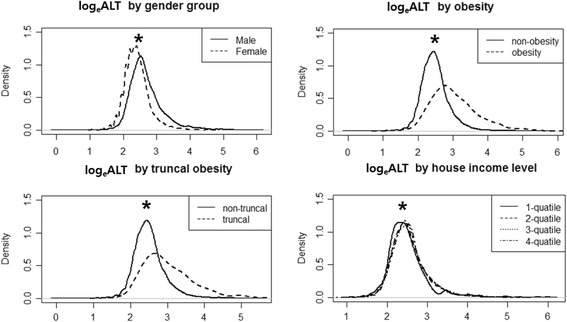
Log-transformed ALT in adolescents by risk factor (* *P* < 0.05)

Log_e_ALT was associated with male sex, obesity, truncal obesity, older age group, and high household income level (Fig. 3). In the univariate regression analysis, log_e_ALT was significantly associated with male sex, obesity, truncal obesity and high household income level. After adjusting for age and sex in the multiple regression analysis, these factors remained significantly associated with log_e_ALT (Table 3). Neither maternal nor paternal education level was significantly associated with log_e_ALT. Multivariate logistic regression analyses of the subgroups showed a gender disparity in risk factor; urban residence was significant only for the female subgroup (OR = 2.6; 95% CI, 1.2-5.9; *P* = 0.0174).

**Table 3:** Regression analysis by log-transformed alanine aminotransferase

	Univariate		Age, sex adjusted		Multivariate	
Variable	Exp (B)	p	Exp (B)	P	Exp (B)	*p*-value
Sex (M vs F)	0.29 (0.26~ 0.31)	< 0.001	0.29 (0.26~ 0.31)	< 0.001	0.26 (0.24~ 0.29)	< 0.001
Age (year)	0.00 (−0.01~ 0.00)	0.8247	0.00 (−0.01~ 0.00)	0.6493	–	–
Town	0.03 (−0.01~ 0.06)	0.1703	0.02 (−0.01~ 0.06)	0.3297	–	–
Obesity	0.57 (0.52~ 0.62)	< 0.001	0.54 (0.49~ 0.58)	< 0.001	0.43 (0.37-0.49)	< 0.001
Truncal obesity	0.48 (0.43~ 0.54)	< 0.001	0.50 (0.45~ 0.55)	< 0.001	0.16 (0.09-0.22)	< 0.001
Income (quartile)	0.03 (0.02-0.04)	< 0.001	0.03 (0.01-0.04)	< 0.001	0.03(0.01-0.04)	< 0.001
Edu^a^ F (quartile)	0.00 (−0.03~ 0.03)	0.9647	0.00 (−0.03~ 0.03)	0.9926	–	–
M (quartile)	0.00 (−0.03~ 0.02)	0.7972	0.00 (−0.03~ 0.02)	0.8636	–	–

^a^Parental education level was included in the KNHANES protocol starting in 2007

## Discussion

The aim of this study was to examine the prevalence, time trends and risk factors of elevated ALT level, a surrogate marker of NAFLD, among Korean children aged 10–18 years from 2001 to 2014. Our major findings were as follows. First, in the study population, the prevalence of elevated ALT levels was 5.3% (S.E. 0.3), and this prevalence plateaued from 2001 to 2014 in adolescents of both genders. Second, obesity, male sex, truncal obesity and high household income level were associated with elevated ALT. Taken together, our findings suggest that trends in the prevalence of elevated ALT are strongly associated with obesity and that this risk factor could be utilized to identify adolescents with NAFLD, thus potentially preventing disease progression at an early age.

Our first major finding is that the prevalence of an elevated ALT level, which indicates suspected NAFLD, was 5.3% (S.E. 0.3) in the study population. ALT ≥30 U/L is a surrogate marker of NAFLD that has been closely correlated with hepatic fat accumulation in obese children based on ultrasonographic findings [7]. To the best of our knowledge, this is the first estimate of NAFLD prevalence using ALT cut-off level of 30 U/L in a Korean general adolescent population. The prevalence of an elevated ALT level differed from that found in other studies in other countries using the same cut-off level. In the NHANES (National Health and Nutrition Examination Survey), the prevalence of elevated ALT levels, defined as > 30 U/L, was 2.3% (1994 to 1998), 8.0% (1999 to 2004), and 6.9% (2007 to 2010) among 12- to 19-year-old adolescents [5]. For Japanese junior high school students, prevalences of 1.6% (2004) and 2.1% (2007) [10] have been reported. Possible explanations for these differences include race, sample size and study years. There was no statistical evidence from the meta-regression that the NAFLD prevalence differed by diagnostic method (ALT versus ultrasonography) [1]. However, elevated ALT levels may overestimate NAFLD in normal-weight children and underestimate it in obese children [11]. The actual prevalence of NAFLD in Korean adolescents would be higher than the prevalence estimated using the elevated ALT level; the prevalence of histologically proven NAFLD in adolescents (11.3 to 17.3%) was higher than that reported in the NHANES study (8%) with a five-year overlap [12].

The trend in the prevalence of elevated ALT plateaued from 2001 to 2014 in adolescents of both genders. A meta-analysis revealed that the prevalence of NAFLD in children and adolescents has not changed over time [1]. As expected, these results are consistent with a study that reported a plateau in childhood obesity, which is closely associated with NAFLD [13]. The prevalence of childhood obesity was stable in Korea between 2001 and 2011, ranging from 7.8 to 7.7% among girls and 7.9 to 8.3% among boys. This finding could be explained by an awareness of the obesity epidemic in children and efforts at both the personal and public levels. The prevalence in each NHANES was 9.1, 10.4, and 10.7% in NHANES IV, V, and VI, respectively. These trend analysis results have some limitations, specifically the possibility of bias resulting from the initial upward trend and subsequent downward trend. Further longitudinal studies based on general populations are needed to examine trends in the prevalence of elevated ALT levels in adolescents.

Our second major finding was that obesity, truncal obesity, male sex and high household income level were associated with logeALT. In this study, obesity was the most significant independent risk factor for elevated ALT; this finding is consistent with those of numerous studies [14]. We used KCDC age- and gender-specific BMI values and defined the 95th percentile, or 25 kg/m^2^, as the cut-off value for “obesity”; this cut-off is lower than other criteria, such as those reported in CDC 2000 (Centers for Disease Control and Prevention Criteria, 2000). Asians have a higher body fat percentage than Europeans with the same BMI and an associated increase in health risk at lower BMI values [15].

The second purpose of this study was to identify risk factors, especially those that are non-invasive. Most children with obesity (75.8%, 717 of 946) did not have elevated ALT, suggesting that obesity and NAFLD are not interchangeable [4]. The correlation between suspected NAFLD and truncal obesity supports previous guidelines indicating that truncal obesity is an aspect of metabolic syndrome and indicates that physicians should check a child’s waist circumference when their BMI is at the 85th percentile or higher [16, 17].

Previous studies have demonstrated that the prevalence of NAFLD varies according to sex and age in both paediatric and adult populations [11]. NAFLD is more common in boys than in girls in both the general population and in clinical studies. The gender disparity in our study is consistent with previous findings, especially those of studies using ALT to assess NAFLD [14]. Although the reason for this remains unclear, differences in muscle mass and sex hormones between genders might be involved [18]. In addition, several studies have concluded that gender-specific normal limits for ALT should be applied [6, 18, 19], but the prevalences based on gender-specific ULNs were similar in our study.

Differences in the prevalence of elevated ALT may also be influenced by environmental factors, location of residence and socioeconomic status [20]. A recent review suggested that the prevalence of NAFLD is higher in countries with a higher economic status and is higher in urban settings than in rural settings [21]. However, there have been few studies on these associations in a general paediatric population. The present study demonstrated that a high household income level was associated with log_e_ALT in the general paediatric population, and a gap between urban and rural populations was for the female adolescents. The results are partially consistent with a recent Korean study that reported that childhood obesity is more prevalent in boys with a high household income [22]. Income has been positively associated with BMI among men in developed Asian countries (Korea and Japan) [23]. It could be explained by gender specific culture and health behaviour. However, these findings are in contrast with those of Fraser et al., who reported that socioeconomic status is inversely associated with elevated ALT levels based on the NHANES 1999–2004 data [7]. Further studies based on the latest data are needed to examine the association between socioeconomic status and NAFLD in adolescents. The urban/rural gap in girls may be explained by lifestyle as less physical activity and a higher calorie diet are more common in urban environments [24]. We also analysed the association between parental education and elevated ALT but found no relationship.

The present study has several strengths. To the best of our knowledge, this is the first study to analyse trends in the prevalence of elevated ALT in Korea or outside the US. The main strength of this study is the relatively large sample size based on five sets of KNHANES data collected within the last 14 years. It is one of the largest epidemiological studies to determine the prevalence of and risk factors for elevated ALT in a general paediatric population. Furthermore, we conducted the analysis using stratification, clustering and sample weights, which are commonly used when analysing the KNHANES data to calculate correct standard errors [25]. In addition, this is the first study in a general population to analyse the association between paediatric ALT elevation and socioeconomic factors, including household income level and parental education level.

Our study has some limitations. First, in 2007, the KNHANES became an annual survey; thus, the methods used in the surveys were inconsistent, and each KNHANES wave was conducted with different subjects. Therefore, sampling bias may have been introduced, especially when estimating the prevalence and trend of elevated ALT levels. Second, we could not exclude all causes of ALT elevation, such as hepatotoxic medications, daily alcohol ingestion and chronic diseases, because the survey questions for children are limited compared with those for adults. However, the KNHANES is the only available nationwide dataset that represents the health status of the general population.

## Conclusions

In summary, this study found that the prevalence of elevated ALT levels was stable from 2001 to 2014 in adolescents of both genders and identified risk factors associated with childhood ALT elevation. These findings suggest that trends in the prevalence of elevated ALT are associated with trends in obesity, and further studies are needed to identify risk factors for paediatric NAFLD.

## Abbreviations

ALT: Alanine aminotransferase
BMI: Body mass index
CDC: Korean Centers for Disease Control and Prevention
CI: Confidence interval
KNHANES: Korean National Health and Nutrition Examination Survey
log_e_ALT: Log-transformed ALT level
NAFLD: Non-alcoholic fatty liver disease
NHANES: National Health and Nutrition Examination Survey
OR: Odds ratio
ULN: Upper limit of normal

## References

[1] Anderson EL, Howe LD, Jones HE, Higgins JP, Lawlor DA, Fraser A (2015). The prevalence of non-alcoholic fatty liver disease in children and adolescents: a systematic review and meta-analysis. PLoS One.

[2] Moran JR, Ghishan FK, Halter SA, Greene HL (1983). Steatohepatitis in obese children: a cause of chronic liver dysfunction. Am J Gastroenterol.

[3] Kohli R, Sunduram S, Mouzaki M, Ali S, Sathya P, Abrams S, Xanthakos SA, Vos M, Schwimmer JB (2016). Pediatric nonalcoholic fatty liver disease: a report from the expert committee on nonalcoholic fatty liver disease (ECON). J Pediatr.

[4] Schwimmer JB (2016). Clinical advances in pediatric nonalcoholic fatty liver disease. Hepatology.

[5] Welsh JA, Karpen S, Vos MB (2013). Increasing prevalence of nonalcoholic fatty liver disease among United States adolescents, 1988-1994 to 2007-2010. J Pediatr.

[6] Park SH, Park HY, Kang JW, Park J, Shin KJ (2012). Aminotransferase upper reference limits and the prevalence of elevated aminotransferases in the Korean adolescent population. J Pediatr Gastroenterol Nutr.

[7] Fraser A, Longnecker MP, Lawlor DA (2007). Prevalence of elevated alanine aminotransferase among US adolescents and associated factors: NHANES 1999-2004. Gastroenterology.

[8] Kweon S, Kim Y, Jang MJ, Kim Y, Kim K, Choi S, Chun C, Khang YH, Oh K (2014). Data resource profile: the Korea National Health and nutrition examination survey (KNHANES). Int J Epidemiol.

[9] Moon JS, Lee SY, Nam CM, Choi J-M, Choe B-K, Seo J-W, Oh K, Jang M-J, Hwang S-S, Yoo MH (2008). 2007 Korean National Growth Charts: review of developmental process and an outlook. Korean J Pediatr.

[10] Tsuruta G, Tanaka N, Hongo M, Komatsu M, Horiuchi A, Hamamoto K, Iguchi C, Nakayama Y, Umemura T, Ichijo T, Matsumoto A, Yoshizawa K, Aoyama T, Tanaka E (2010). Nonalcoholic fatty liver disease in Japanese junior high school students: its prevalence and relationship to lifestyle habits. J Gastroenterol.

[11] Nobili V, Alisi A, Newton KP, Schwimmer JB (2016). Comparison of the phenotype and approach to pediatric vs adult patients with nonalcoholic fatty liver disease. Gastroenterology.

[12] Schwimmer JB, Deutsch R, Kahen T, Lavine JE, Stanley C, Behling C (2006). Prevalence of fatty liver in children and adolescents. Pediatrics.

[13] Bahk J, Khang YH (2016). Trends in measures of childhood obesity in Korea from 1998 to 2012. J Epidemiol.

[14] Fusillo S, Rudolph B (2015). Nonalcoholic fatty liver disease. Pediatr Rev.

[15] Quak SH, Furnes R, Lavine J, Baur LA, Obesity Working G (2008). Obesity in children and adolescents. J Pediatr Gastroenterol Nutr.

[16] Barlow SE, Expert C (2007). Expert committee recommendations regarding the prevention, assessment, and treatment of child and adolescent overweight and obesity: summary report. Pediatrics.

[17] Vajro P, Lenta S, Socha P, Dhawan A, McKiernan P, Baumann U, Durmaz O, Lacaille F, McLin V, Nobili V (2012). Diagnosis of nonalcoholic fatty liver disease in children and adolescents: position paper of the ESPGHAN hepatology committee. J Pediatr Gastroenterol Nutr.

[18] Poustchi H, George J, Esmaili S, Esna-Ashari F, Ardalan G, Sepanlou SG, Alavian SM (2011). Gender differences in healthy ranges for serum alanine aminotransferase levels in adolescence. PLoS One.

[19] Schwimmer JB, Dunn W, Norman GJ, Pardee PE, Middleton MS, Kerkar N, Sirlin CB (2010). SAFETY study: alanine aminotransferase cutoff values are set too high for reliable detection of pediatric chronic liver disease. Gastroenterology.

[20] Berardis S, Sokal E (2014). Pediatric non-alcoholic fatty liver disease: an increasing public health issue. Eur J Pediatr.

[21] Zhu JZ, Dai YN, Wang YM, Zhou QY, Yu CH, Li YM (2015). Prevalence of nonalcoholic fatty liver disease and economy. Dig Dis Sci.

[22] Bahk J, Khang YH (2016). Trends in childhood obesity and central adiposity between 1998-2001 and 2010-2012 according to household income and urbanity in Korea. BMC Public Health.

[23] Murayama N (2015). Effects of socioeconomic status on nutrition in Asia and future nutrition policy studies. J Nutr Sci Vitaminol (Tokyo).

[24] Song Y, Wang HJ, Dong B, Ma J, Wang Z, Agardh A (2016). 25-year trends in gender disparity for obesity and overweight by using WHO and IOTF definitions among Chinese school-aged children: a multiple cross-sectional study. BMJ Open.

[25] Kim Y, Park S, Kim NS, Lee BK (2013). Inappropriate survey design analysis of the Korean National Health and nutrition examination survey may produce biased results. J Prev Med Public Health.

